# Greater Risk of Negative Health Outcomes of Older Adults Living Alone in Vietnam: A Community Survey

**DOI:** 10.3390/ijerph182111115

**Published:** 2021-10-22

**Authors:** Man Thi Hue Vo, Keiko Nakamura, Kaoruko Seino, Thang Van Vo

**Affiliations:** 1 Department of Global Health Entrepreneurship, Tokyo Medical and Dental University, Tokyo 113-8519, Japan; manvo.ith@tmd.ac.jp (M.T.H.V.); seino.ith@tmd.ac.jp (K.S.); 2 WHO Collaborating Centre for Healthy Cities and Urban Policy Research, Tokyo 113-8519, Japan; 3 Faculty of Public Health, University of Medicine and Pharmacy, Hue University, Hue 530000, Vietnam; vovanthang147@hueuni.edu.vn; 4 The Institute for Community Health Research, University of Medicine and Pharmacy, Hue University, Hue 530000, Vietnam

**Keywords:** older adult, living alone, health, outcome

## Abstract

In modern Asian societies, there has been a shift in the living arrangements of older adults away from living with others. Knowing the health characteristics of individuals living alone can help identify high-risk groups. This cross-sectional study aimed to describe characteristics of the Vietnamese older adults and to investigate the association between living alone and their reported health outcomes by utilizing survey data of individuals aged ≥60 years in Vietnam in 2018. The community survey included questions about sociodemographic factors, living arrangement, and self-reported physical functional status. Multivariate logistic regression was used to examine whether or not living alone was a predictor of health outcomes. Of 725 study participants, 8.9% lived alone. These participants were more likely to be female, aged 70–79 years, living in rural areas, and currently single or previously married. After adjusting for covariates, older adults who were living alone were more likely to have arthritis (adjusted odds ratio [AOR] = 1.95, 95% confidence interval [CI]: 1.10–3.45), a history of falling (AOR = 2.44, 95% CI: 1.02–5.82), visual difficulties (AOR = 1.89, 95% CI: 1.04–3.41), feelings of loneliness (AOR = 1.95, 95% CI: 1.10–3.47), and high fear of falling (AOR = 1.88, 95% CI: 1.02–3.46). Older adults living alone in Vietnam were at greater risk of negative health consequences than those living with others. Screening and providing adequate social support for this specific population is important in preventing the adverse effects of solitary living among these older adults.

## 1. Introduction

The population of Asia is rapidly aging due to the rapid decline in birth rate and rising life expectancy. Among this, Vietnam is one of the countries with fastest ageing rate in the world [1]. The proportion of older adults was reported to be 12% of total Vietnamese population in 2019 [2].

Consequently, living alone has become an increasingly common living arrangement along with the changes towards population aging and household composition [3]. Similarly, Vietnam is experiencing many changes in urbanization and family structure. A national survey conducted in 2020 reported that 8.6% of Vietnamese older adults were living alone, which was higher than the estimate reported in the first national aging data in 2011 (6.2%) [4,5]. This prevalence was comparable to recent statistics from other countries in the Southeast Asian region, such as Singapore (7.9%) [6], Malaysia (10.1%) [7] and Thailand (13.4%) [8].

Past literatures have explored the diverse impact of living alone on the health and well-being of older people. In studies, living arrangement is often categorized as living alone if the respondent was living by oneself in the household. Living alone was reported to be at higher risk of negative health consequences, particularly deterioration in cognitive function, chronic conditions, worsen mental health, activities of daily living, risk for falls, and social isolation among community-dwelling older adults in Europe [9,10], UK [11] and Asian countries such as the Philippines [12] and Indonesia [13]. However, in some European settings, the negative impact of living alone on health outcomes no longer remained when loneliness factor was considered [10]. On the other hand, the positive health outcomes associated with living alone were reported in several settings as well. Some studies presented that older adult who lived alone had improved cognitive ability, physical and functional status in Europe [10], Singapore [6], UK [14] and higher social participation in Vietnam and Thailand [15]. Understanding how living arrangements influences older people’s health and well-being is helpful for public health and social welfare and allows health organizations to support this population in maintaining living in the community. 

Compared to the research evidence for living arrangements among older adults in the community from developed settings, studies in developing countries in Asia are found to be scarcer. Recently, there have been few studies in South-East Asian countries, which share similar characteristics with Vietnam, report inconsistent findings about the association between living arrangement and health outcomes such as cognitive ability in Singapore and Thailand [6,16], or psychological health in Indonesia, Vietnam, Thailand and Myanmar [15,17]. For instance, living with others may play a positive role in maintaining cognitive function for Thailand older man [16], but not for Singapore community-dwelling older adults [6]. There is a need for further evidence on living arrangement status in this region. 

The impact of living alone on health outcomes could depend on certain social environments and existing healthcare services. In Southeast Asian societies such as Vietnam, living alone may be perceived as an undesirable living arrangement by relating to negative consequences for the care of older people [18]. This can be explained by some reasons. Many developed countries are already ahead low- and middle-income countries in implementing formal long term care system due to the health and social services demand of older people and reduced availability of informal caregivers [19]. For example, Japan, the world’s super-aged society, has public long-term care insurance system since 2000, which aims to help older people including those who are solo-living, can live independently in their own homes [20]. Meanwhile, in Vietnam, the public health system has yet to have a national long-term care. Although social protection centers that accommodate lonely and poor older persons with care and nursing services have been established, the issues related to social exclusion of these vulnerable groups have still emerged [21]. As well, there was a lack of regulations and support from the related authorities in planning for nursing homes or senior care facilities [21]. This situation can be viewed as similar to other Southeast Asian countries, where the demand for older people’s care was rapidly rising but the countries had not reached the level of economic development needed for public care services [18]. Another explanation is that older Asian people is commonly taken care of informally and by family members [18]. In particular, Vietnamese older people are unfamiliar with living in institutional care [22]. Moreover, under the Confucian principles, Vietnamese people are known to be more community dependent that emphasize family and community connections. A recent study confirmed this by reporting the preferred choice of living arrangement of Vietnamese older adults, that 74% desired to live amongst or near to their children [4]. Hence, older adults living alone in Vietnam may be particularly more vulnerable to health risks. 

Given the increasing rate of population ageing, change in family structure and composition, combined with the above-mentioned factors that influence the relationship between living alone and health outcomes, we assume that older Vietnamese people living alone are more likely than those who lived with others to experience negative physical and mental health consequences. Therefore, this study adds to the literature by assessing the association between living alone and health outcomes, presented by chronic diseases, falls history, functional health status and subjective well-being among community-dwelling older adults in Vietnam.

## 2. Materials and Methods 

### 2.1. Setting and Participants

A cross-sectional study of community-dwelling adults residing in Hue City (urban) and Phu Vang district (rural) of the Thua Thien Hue Province, Vietnam, was conducted between June and July 2018. 

According to the sample size formula in prevalence studies, 730 participants were recruited using a two-stage random cluster sampling method. In the first stage, two units among 27 units in the urban area and three units out of 20 units in the rural area were randomly selected. Secondly, from each selected unit, 146 households with people aged 60 years old or over were randomly chosen. This sample was geographically representative with data collected in both the urban and rural areas.

Face-to-face interviews using structured questionnaires were used to collect the data. The data collectors were trained healthcare providers who majored in public health. Written, informed-consent was taken prior to participation and those who refused or were unable to speak were excluded from this study. The final sample size used for data analysis was 725, with a survey response rate of 99%. 

### 2.2. Measurements

#### 2.2.1. Living Arrangement

The participants were asked “Who are you living/residing with?” during the interview. The responses were classified as “living alone” or “with others”. 

#### 2.2.2. Outcome Variables 

The variables that measured health outcomes were categorized into selected chronic diseases and fall history, functional health status, and subjective well-being assessment. Self-reported chronic diseases were asked and checked with the previous information in participants’ medical documents, included arthritis and hypertension due to their considerably high prevalence in this study population. Fall history was assessed by the responses to the question, “In the last 12 months, has there been any event where you suffered from bodily injuries?”. Functional health status included cognitive and visual abilities. Cognitive function was evaluated using the Mini-Mental State Examination; impairment was defined as a score of <23 points. Visual ability was classified as “no difficulty” and “having some difficulty”, which was a combination of self-rated responses including “difficult”, “very difficult”, and “unable to perform”.

Subjective well-being was elicited by perceived social support, fear of falling (FOF), and a single yes/no response to the question, “In the past month, did you ever feel very lonely or distant from others?”, which was later coded as “feeling very lonely” in the results. Social support was measured using the Multidimensional Scale of Perceived Social Support. Mean total scores of 1.0–5.0 points represent low and moderate levels of support, and those of 5.1–7.0 points represent high levels of support. FOF was assessed using the Falls Efficacy Scale—International. Scores of 16–19, 20–27, and 28–64 points represent low, moderate, and high levels of concern, respectively. 

Age group, gender, residential area, and marital status were selected as covariates for the associations between living arrangement and health outcome variables. 

### 2.3. Ethics, Approval, and Informed Consent

The procedures in this study were in accordance with the ethical standards of the Ethical Review Committee of University of Medicine and Pharmacy, Hue University, Vietnam.

Written, informed-consent was obtained from all participants or their guardian prior to participation in this study. 

### 2.4. Data Analysis

Descriptive characteristics were stratified according to the living arrangement status. The association between the participants’ characteristics and the living arrangement was assessed using the Chi-square test. Health outcomes were the dependent variables. Multivariate logistic regression analysis, adjusted for gender, age, residential area, and marital status, was used to examine the association between living alone and the health outcome variables.

All statistical analyses were performed using SPSS version 22 (IBM, Armonk, NY, USA). Statistical significance was indicated by *p*-values of <0.05.

## 3. Results

A total of 725 participants were included in this study. The mean age was 72 ± 8.8 years and 8.9% of the participants (*n* = 65) lived alone. This sample proportionally reflects specified characteristics of the Vietnamese national population in terms of gender, age group, and educational level [5].

### 3.1. Characteristics of Older Adults According to Their Living Arrangement

The participants who lived alone were likely to be women and in the 70–79 age group. These participants were more likely than their counterparts to live in a rural area, be currently single or previously married, and have a history of falls, arthritis, and visual difficulties. They were also more likely to have a high level of social support, normal cognitive function, FOF, and loneliness (Table 1).

### 3.2. Association between Living Arrangement and Reported Chronic Diseases and Fall History

Living alone was associated with arthritis (adjusted odds ratio [AOR] = 1.95, 95% confidence interval [CI]: 1.10–3.45) and a history of falling (AOR = 2.44, 95% CI: 1.02–5.82). No association was observed between living alone and hypertension (Table 2).

### 3.3. Association between Living Arrangement and Reported Functional Health Status

Older adults who lived alone were more likely than their counterparts to have cognitive impairment (OR = 2.02, 95% CI: 1.13–3.62). After adjusting for covariates, a significant association was found between living alone and visual difficulties (AOR = 1.89, 95% CI: 1.04–3.41) (Table 2).

### 3.4. Association between Living Arrangement and Subjective Well-Being

Older adults who lived alone were more likely than were their counterparts to have a low and moderate level of social support (OR = 2.15, 95% CI: 1.21–3.81). After adjusting for covariates, a significant association was found between living alone and feeling very lonely (AOR = 1.95, 95% CI: 1.10–3.47) and having a high FOF (AOR = 1.88, 95% CI: 1.02–3.46). No association between living arrangements and low and moderate social support was found (Table 2).

## 4. Discussion

This study found that almost three quarters (47/65, 72.3%) of older persons living alone were female, and approximately 70% (45/65) of those living alone were 70 years and above. The community-dwelling older adults living alone were more likely than those living with others to report having arthritis, a history of falling, visual difficulty, feeling very lonely, and high FOF. 

Our analysis revealed an association between living arrangements and chronic diseases, represented by arthritis in this population. Similar association between living alone and arthritis were reported from a higher income setting [11]. In addition, a previous study confirmed that people with arthritis experienced more loneliness and less social support [23], which may explain the association between living alone and arthritis in our study. Our findings are also consistent with earlier studies, which have found positive associations between living alone and history of falling [11,24]. Chronic conditions such as arthritis reported more frequently by those living alone in our study may explain the increased likelihood of a fall history in this group. The impact of living arrangements on arthritis, and fall-related accidents should be carefully considered.

This study did not detect any association between living alone and cognitive impairment in the adjusted model. This finding was in agreement with previous findings [25,26], however, disagreed with some studies that those living alone were more likely to report poorer cognitive ability [11,27]. These inconsistencies may be accounted for by the differences in cognitive function measurements across different settings, and the associations between living alone and cognitive impairment could be more evident in follow-up studies. Our findings implied that it could be social isolation or lack of interaction even when living with others, rather than the living arrangement itself that associate with cognitive decline. Additional analysis from our data also observed that the mean cognitive score of the participants with low-moderate perceived social support levels was significantly lower than those with a high perceived level of social support. (See Appendix A, Table A1). However, more longitudinal studies to investigate on these associations are much needed for clarification. 

Unexpectedly, in the present study, older adults living alone did not report having a lower level of social support than that reported by individuals living with others; nevertheless, they were more likely to feel lonely or distant, which was in agreement with a study from Singapore [27]. These associations were independent of age, gender, residential area, and marital status. Evidence from previous reports in both higher income society in the US [28] and similar Asian settings [17,27,29] have already highlighted that individuals living alone were lonelier, but had more than [14,27] or as much social engagement as those living with others in Thailand and our study setting, Vietnam [15]. The loneliness faced by community-dwelling older adults could be due to the social stigma of living alone, as well as perceived expectations from Asian communities. 

The findings that living alone was significantly associated with visual impairment can be explained by the presence of loneliness. In this study, a majority of the participants who were living alone felt very lonely (55.4%), which was found to be prevalent among older people with visual impairment [30]. Although the relationship of cause and effect could not be determined by this cross-section study, this result indicated that attention should be given to the visual ability of solitary-living older adults in Vietnam when developing interventions because of the high rate of loneliness among this group. 

Community-dwelling older adults living alone in our study were more than twice as likely to experience high FOF in the study, compared to their counterparts. This result was confirmed by previous study [29] that alone older adults experienced more FOF than the other groups. This could be because majority of the participants living alone in our study were female, which has been commonly reported as predictor of FOF [31]. Besides, living alone was stated to strongly associated with falls risks [24]. This finding may suggest the greater care needs of older persons living alone in light of fall prevention. 

This study found that there were 8.9% community-dwelling older adults living alone, which was similar to the most recent statistics from the Vietnam national survey in 2020 (8.6%) [4]. The increase in the proportion of older persons living alone in Vietnam compared to the results from the national survey in 2011 (6.2%) [5] illustrates the changes in the living arrangement of older adults in Vietnam, which result from socio-economic development, population aging, and modern family structure. In agreement with Vietnam national survey report [5], our study found higher proportion of solitary-living older persons were women and were living in rural residential areas, which was also consistent with findings from other Asian countries such as the Philippines [12], Myanmar, and Thailand [15]. Current social welfare policy and health care systems should be expanded and redesigned to meet the need of this growing vulnerable population in areas with less availability of healthcare delivery. 

Our present results indicated that older adults living alone are vulnerable to health problems. While living separately may be protective in some settings due to healthcare system accessibility, well-established long-term health services, and a network of social activities for seniors, similar amenities are not widely available in Vietnam yet, which may in turn have negative influence on solitary-living population.

The advantage of this study was the large representative sample of Vietnamese elderly and the use of standardized scales to measure the outcome variables. However, there are several limitations to this study. As a cross-sectional study, the causation direction cannot be inferred and the results should be interpreted with caution. Although our sample is representative of Vietnam’s older adults, the results may not be generalized to other populations with different contexts and demographics. Further longitudinal studies are needed to better understand the risk factors associated with living alone. 

## 5. Conclusions

This study has shown that living alone may increase the risk of negative health outcomes among older adults in Vietnam, including arthritis, a history of falls, visual difficulties, loneliness, and FOF. These health issues should be carefully addressed regardless of age, gender, residence location, or marital status. An age-friendly environment and good social connections are beneficial for community-dwelling older adults. The present findings can be relevant to health service providers, local authorities, and communities in Vietnam and other similar Asian settings, where preventing health problems in community-dwelling older adults is important. Future studies should focus on evaluating the impact of living arrangements on FOF in Asia.

## Figures and Tables

**Table 1:** Baseline characteristics of older adults according to their living arrangement.

Characteristics of Older Adults		All (*n* = 725)	Living Alone (*n* = 65)	Living with Others (*n* = 660)	*p*-Value
			*n* (%)	*n* (%)	
Gender	Female	423	47 (72.3)	376 (57.0)	0.017
	Male	302	18 (27.7)	284 (43.0)	
Age group, years	60–69	340	20 (30.8)	320 (48.5)	0.006
	70–79	209	29 (44.6)	180 (27.3)	
		176	16 (24.6)	160 (24.2)	
Residential area	Rural	282	34 (52.3)	248 (37.6)	0.020
	Urban	443	31 (47.7)	412 (62.4)	
Marital status	Single or previously married	201	52 (80.0)	149 (22.6)	<0.001
	Married	524	13 (20.0)	511 (77.4)	
History of falling	Yes	54	10 (15.4)	44 (6.7)	0.011
	No	671	55 (84.6)	616 (93.3)	
Arthritis	Yes	243	31 (47.7)	212 (32.1)	0.011
	No	482	34 (52.3)	448 (67.9)	
Hypertension	Yes	318	36 (55.4)	282 (42.7)	0.050
	No	407	29 (44.6)	378 (57.3)	
Visual ability	Have difficulty	355	43 (66.2)	312 (47.3)	0.004
	No difficulty	370	22 (33.8)	348 (52.7)	
Walking ability	Have difficulty	222	43 (66.2)	460 (69.7)	0.554
	No difficulty	503	22 (33.8)	200 (30.3)	
Level of social support	Low and moderate	423	17 (26.2)	285 (43.2)	0.008
	High	302	48 (73.8)	375 (56.8)	
Cognitive function	Impairment	123	18 (27.7)	105 (15.9)	0.016
	Normal	602	47 (72.3)	555 (84.1)	
Level of fear of falling	High	296	39 (60.0)	257 (38.9)	0.001
	Not high	429	26 (40.0)	403 (61.1)	
Feel very lonely or distant from other people	Yes	218	36 (55.4)	182 (27.6)	<0.001
	No	507	29 (44.6)	478 (72.4)	

**Table 2:** Association between living arrangement and self-reported health outcomes (*n*= 725).

	Arthritis	Hypertension	Fall History	Cognitive Impairment	Visual Difficulty	Low/Moderate Social Support	Feel Very Lonely	High Fear of Falling
***Crude odd ratio** (95% CI)*								
**Living arrangement**								
Living with others	1	1	1	1	1	1	1	1
Living alone	1.93	1.66	2.55	2.02	2.18	2.15	3.26	2.35
	(1.15–3.22) ^a^	(1.00 – 2.78)	(1.22–5.34) ^a^	(1.13–3.62) ^a^	(1.28–3.73) ^b^	(1.21–3.81) ^b^	(1.94–5.47) ^c^	(1.40–3.96) ^b^
***Adjusted odds ratio **^1^ (95% CI)*								
**Living arrangement**								
Living with others	1	1	1	1	1	1	1	1
Living alone	1.95	1.70	2.44	1.28	1.89	1.47	1.95	1.88
	(1.10–3.45) ^a^	(0.97–2.98)	(1.02–5.82) ^a^	(0.65–2.55)	(1.04–3.41) ^a^	(0.79–2.75)	(1.10–3.47) ^a^	(1.02–3.46) ^a^
**Gender**								
Male	1	1	1	1	1	1	1	1
Female	2.31	0.95	2.97	1.97	1.33	1.33	1.45	3.41
	(1.63–3.29) ^c^	(0.69–1.31)	(1.45–6.10) ^b^	(1.22–3.20) ^b^	(0.96–1.84)	(0.96–1.84)	(1.00–2.10) ^a^	(2.36–4.94) ^c^
**Age group**								
60–69 years	1	1	1	1	1	1	1	1
70–79 years	1.38	1.43	2.10	2.09	1.40	1.62	1.07	2.08
	(0.95–2.02)	(1.00–2.03) ^a^	(1.05–4.20) ^a^	(1.19–3.66) ^a^	(1.19–3.66)	(1.12–2.32) ^a^	(0.72–1.61)	(1.41–3.06) ^c^
≥80 years	1.30	1.58	2.00	6.96	3.02	1.48	1.51	5.98
	(0.85–2.00)	(1.07–2.32) ^a^	(0.93–4.29)	(4.03–12.00) ^c^	(2.01–4.52) ^c^	(1.00–2.21)	(0.98–2.31)	(3.85–9.31) ^c^
**Residential area**								
Urban	1	1	1	1	1	1	1	1
Rural	0.82	0.80	0.59	2.11	1.65	1.16	1.27	1.70
	(0.59–1.14)	(0.59–1.10)	(0.31–1.12)	(1.37–3.26) ^b^	(1.20–2.25) ^b^	(0.85–1.58)	(0.90–1.80)	(1.21–2.39) ^b^
**Marital status**								
Married	1	1	1	1	1	1	1	1
Single or previously married	0.78	0.94	0.79	1.48	1.01	1.55	2.22	0.94
	(0.52–1.18)	(0.63–1.39)	(0.39–1.61)	(0.89–2.44)	(0.67–1.51)	(1.03–2.34) ^a^	(1.47–3.35) ^c^	(0.62–1.43)

^1^: Adjusted for gender, age, residential area, and marital status. ^a^*p* < 0.05; ^b^ *p* < 0.01, ^c^ *p* < 0.001.

## Data Availability

The dataset in the current study is available from the corresponding author on reasonable request.

## Appendix A

**Table A1:** Mean MMSE score of older adults of low–moderate social support level and high social support level.

	Low-Moderate Social Support Level (*n* = 423)	High Social Support Level (*n* = 302)	*p*	95% CI	
				Lower	Upper
MMSE mean score	25.46	26.59	0.003	−1.90	−0.38
